# Wild Fruits of *Crataegus monogyna* Jacq. and *Sorbus aria* (L.) Crantz: From Traditional Foods to Innovative Sources of Pigments and Antioxidant Ingredients for Food Products

**DOI:** 10.3390/foods12122427

**Published:** 2023-06-20

**Authors:** Cristina Tamayo-Vives, Patricia García-Herrera, María Cortes Sánchez-Mata, Rosa M. Cámara-Hurtado, María Luisa Pérez-Rodríguez, Laura Aceituno, Manuel Pardo-de-Santayana, María Inês Días, Lillian Barros, Patricia Morales

**Affiliations:** 1 Department of Nutrition and Food Science, Faculty of Pharmacy, University Complutense of Madrid, Pza, Ramón y Cajal s/n, 28040 Madrid, Spain; critamay@ucm.es (C.T.-V.); cortesm@ucm.es (M.C.S.-M.); rm.camara@ucm.es (R.M.C.-H.); peromalu@ucm.es (M.L.P.-R.); patricia.morales@farm.ucm.es (P.M.); 2 Department of Biology (Botanic), Faculty of Sciences, Autonomous University of Madrid, Cantoblanco Campus, 28049 Madrid, Spain; aceitunomata@yahoo.es (L.A.); manuel.pardo@uam.es (M.P.-d.-S.); 3 Center for Research in Biodiversity and Global Change (CIBC-UAM), Autonomous University of Madrid, 28049 Madrid, Spain; 4 Centro de Investigação de Montanha (CIMO), Instituto Politécnico de Bragança, Campus de Santa Apolónia, 5300-253 Bragança, Portugal; maria.ines@ipb.pt (M.I.D.); lillian@ipb.pt (L.B.); 5 Laboratório Associado para a Sustentabilidade e Tecnologia em Regiões de Montanha (SusTEC), Instituto Politécnico de Bragança, Campus de Santa Apolónia, 5300-253 Bragança, Portugal

**Keywords:** peel, epidermis, anthocyanins, antioxidant capacity, hydroxybenzoic acids, hydroxycinnamic acids, flavonols, cyanidin-*O*-hexoxide

## Abstract

Hawthorn (*Crataegus monogyna* Jacq.) and whitebeam (*Sorbus aria* (L.) Crantz) are wild species traditionally used as ethnic foods in the Mediterranean area. Their red berries, and mainly the peels, may be used as ingredients due to their color (replacing other synthetic colorants) or functional properties. Some previous studies analyze all edible fruits, but there is very little literature on the composition and properties of the pulpless epidermis of the fruits of *C. monogyna* and no literature concerning the fruits of *S. aria*. Total phenolic compounds (TPC) and families of hydroxybenzoic acids, hydroxycinnamic acids, flavonols, and total monomeric anthocyanins were determined in the epidermis of *C. monogyna* and *S. aria* fruits. The in vitro antioxidant capacity was also determined using QUENCHER (Quick-Easy-New-CHEap-Reproducible) methodology. Anthocyanins profiles were analyzed in hydroalcoholic extracts through HPLC/MS. *C. monogyna* fruits presented higher content of TPC than *S. aria*, with hydroxybenzoic acids (2870.6 mg GAE/100g dw) as the major family, followed by flavonols (771.4 mg QE/100 g dw) and hydroxycinnamic acids (610.3 FAE/100 g dw). Anthocyanins were found in 251.7 mg cyanidin-3-glucoside/100 g dw, characterized by the content of cyanidin-*O*-hexoxide and peonidin-*O*-hexoxide. The levels of these compounds correlated with higher values of a* parameter (higher intensity of reddish color). These fruits also showed higher antioxidant capacity by Q-Folin–Ciocalteu and Q-FRAP. *S. aria* peels had fewer phenolic compounds, particularly anthocyanins (33.7 mg cyanidin-3-glucoside/100 g dw), containing different cyanidin derivatives. From these results, new insights about the composition of the epidermis of these wild fruits are provided, and their potential as ingredients for the food industry is corroborated.

## 1. Introduction

In the Mediterranean basin, there is a great diversity of wild fruits that have been traditionally used for medicinal and culinary purposes. Due to the growing demand for healthy, sustainable, and antioxidant foods, the culinary use of some wild resources is significantly increasing [1]. Traditional fruits have been studied in many ethnobotanical and nutritional studies, especially red berries, due to their taste, color, and sweetness, but also for their nutritional and bioactive properties [2,3]. Many of these properties are due to the presence of different phenolic compounds, such as phenolic acids (hydroxycinnamic and hydroxybenzoic derivatives) and flavonoids (anthocyanins and flavonols), all of them related to their antioxidant potential [4,5,6,7]. The most interesting pigments present in red berries are anthocyanins, which have different coloration depending on the pH.

Hawthorn (*Crataegus monogyna* Jacq.) and whitebeam (*Sorbus aria* (L.) Crantz), belonging to the family *Rosaceae*, subfamily *Maloideae*, are examples of wild species producing red berries with great potential as food ingredients. They are widely distributed in Europe, particularly in the Iberian Peninsula [8,9,10]. The hawthorn berry is red-colored when ripe (summer/autumn), has a size of less than 1 cm, and contains a small seed. Whitebeam fruit is globose, with an acidic taste and consistency, and presents small macules called lenticels, red or brown when ripe in autumn. These fruits have been traditionally used as either raw fruits or in jams, pastries, liquors, or other local products as a part of gastronomy in the Mediterranean area. Due to its potential health-beneficial effects, as well as its antioxidant and antimicrobial activity, hawthorn has recently become important in the food industry [11]. However, there is very little knowledge about the composition of *C. monogyna* fruits from the Iberian Peninsula. *Sorbus* species can also be a valuable base of functional food or value-added food products, but the detailed chemical composition and antioxidant activity of *S. aria* from the Iberian Peninsula, especially in Spain, is very limited or lacking. It must be emphasized that to the authors’ knowledge, there are no composition data of *S. aria* from the Iberian Peninsula until now.

Furthermore, most published studies on the chemical composition of wild berries are focused on the analysis of whole fruits, but the epidermis of fruits has the greatest potential as a food ingredient and has been scarcely studied as separated from the whole fruits. However, in these types of fruits, phenolic compounds are mostly found in the peel of the fruits, contributing to the color as well as antioxidant capacity of the fruits, so the use of their peel would be a good strategy for the formulation of foods and/or food supplements with functional properties. Most reports on the phenolic composition of these fruits were completed using whole fruits [8,10,12], so knowledge about the composition and properties of the epidermis as an ingredient is lacking.

Additionally, the extract of the compounds of interest in hydroalcoholic solutions could be used as food ingredients for their functional properties. In this case, the efficiency of the extraction process would highly influence the presence of the compounds of interest in the final food.

With the purpose of extract characterization, solid/liquid extraction techniques may not be often fully efficient, requiring a high amount of sample and solvent, as well as magnetic stirring, ultrasound, or other techniques to facilitate extraction. The polarity of the compounds of interest also influences the solubility in the extraction solvent. Furthermore, plant materials present lipophilic and hydrophilic molecules, which could be bound to the antioxidant components. For these reasons, it is difficult to obtain high efficiency using only one solvent or mixture of solvents, as antioxidant capacity is frequently underestimated with these measurement methods. Del Pino-García et al., (2015) optimized the QUENCHER procedure (QUick, Easy, New, CHEap, and Reproducible) to evaluate the antioxidant capacity without any extraction when the compounds are bound to the insoluble matrices taking advantage of the surface reaction phenomenon between solid (bound antioxidant compounds) and liquid (soluble free radicals) material [13]. This methodology allows a direct reaction, using very little sample, generating less waste, and contributing to the green chemistry, which is nowadays an important factor to be considered in analytical chemistry from an environmental point of view.

This work aimed to characterize the presence of phenolics in the peel, specifically in the epidermis, of the fruits of *C. monogyna* and *S. aria* with special interest on anthocyanins, with the purpose of revalorizing these fruits as sources of colored antioxidant ingredients for the food industry.

## 2. Materials and Methods

### 2.1. Samples

**Figure 1:**
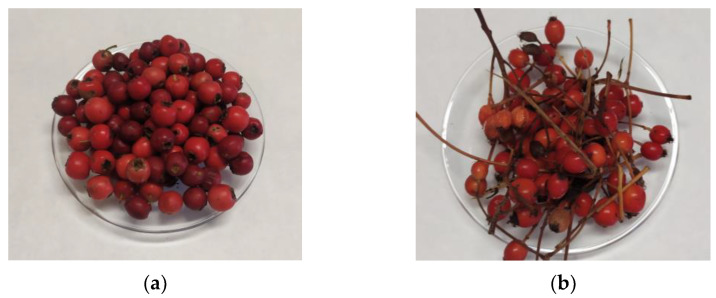
Fruits gathered in their optimal ripening status: (**a**) *Crataegus monogyna* Jacq. fruits; (**b**) *Sorbus aria* (L.) Crantz fruits.

The selected species were identified in their natural habitats in the central part of Spain, and fruits were gathered in their optimal ripening status between September and October 2021, with collection permits Ref. PN-NC_032021 and Ref. ABSCH-IRCC-ES-257749-1 issued by the Ministry of Agriculture, Fisheries and Food, Government of Spain (Figure 1). Each type of fruit was collected in two different locations, from different trees and shrubs, to obtain a representative sample. Then, they were transferred to the laboratory of the Department of Nutrition and Food Science of the UCM (Universidad Complutense de Madrid), refrigerated in transparent plastic bags, and properly identified.

Table 1 shows the data of sites of the samples of *C. monogyna* and *S. aria*. The whole fruit without seed was used for fresh fruit characterization; the rest of the fruit was peeled and processed for freeze-drying, being crushed as soon as possible to obtain a fine powder (sieved through 425 μm) and preserved at −20 °C in a dark and dry environment.

### 2.2. Methods

#### 2.2.1. Characterization of Fresh Fruits

All determinations in fresh fruits without seeds were made in triplicate for each sample coming from each locality. Firstly, moisture was determined by desiccation in an oven at 105 °C for 24 h until a constant weight was reached (984.25 A.O.A.C., 2006) [14]. Other aliquots of 2.5 g of homogenized fresh fruit were suspended in 25 mL distilled water and left to stand for 10 min, and the supernatant was measured directly for °Brix determination by refractometry at 20 °C (932.14C, A.O.A.C., 2006) in an Atago refractometer [14]. To express the results, a correction was determined due to the dilution made. In the same suspension, pH was measured using a pH meter MicropH–2000 (Crison Instruments, Barcelona, Spain) (981.12 A.O.A.C., 2006) [14].

Titratable acidity (TA) was measured by potentiometry by titration with 0.01 N NaOH to a pH value of 8.1 (942.15, A.O.A.C., 2006) [14]. The result was expressed as milliequivalents (meq) of NaOH to neutralize the acids present in 100 g of fresh sample. Finally, the ripening index was calculated by the ratio between °Brix and titratable acidity.

#### 2.2.2. Analysis of Dried Fruit Epidermis

Freeze-dried fruit epidermis was subjected to analysis of color as well as determination of phenolics, hydroxybenzoic and hydroxycinnamic acids, flavonols, monomeric anthocyanins, and antioxidant capacity through QUENCHER methodology. Analytical procedures are briefly described below.

- Color

Determination of color in dry plant material was carried out following the method described by Vega et al., (2023) using a colorimeter ColorFlex (HunterLab) [15]. Once the material was crushed and sieved, it was placed in a perfectly clean Petri dish. The colorimeter directly provided the L*, a*, and b* parameters. By logging the results in the database EasyGRB, which provided the RGB parameters, the the color result was obtained.

- Q-Total Phenolic Content

The determination of total phenolic content (TPC) in dry plant material was carried out according to the Q-Fast Blue BB methodology described by Palombini et al., (2016) [16].

Each sample of freeze-dried fruit epidermis was weighed (1 ± 0.1 mg) in Falcon tubes, covered with aluminium foil and 0.4 mL of 0.1% fast blue BB, 0.4 mL of 5% NaOH, and 4 mL of distilled water were added; the sample was then stirred in a vortex (Velp Scientifica, Usmate Velate, Italy) and left in orbital agitation for 45 min. Absorbance at 420 nm was read in a microplate with a Synergy HTX multi-mode reader spectrum (Winooski, VT, USA) after centrifugation for 10 min at 6500 rpm and filtration.

The result was expressed as mg of gallic acid equivalent per gram of product (mg GAE/100 g dw) using a calibration line for different quantities of the standard, made from solutions of different concentrations (0–160 µg/mL) of gallic acid with the same treatment as the samples, and left stand in darkness during 1 h at room temperature; measurement of absorbance at 420 nm was read in a Synergy HTX multi-mode reader spectrum using microplates.

- Q-Hydroxybenzoic acids

The determination of hydroxybenzoic acids was adapted from Bonoli et al., (2004) [17]. Triplicate samples of dry plant material were weighed (1 ± 0.1 mg) in Falcon tubes using a precision balance (BOECO, Hamburg, Germany). The tubes were covered with aluminium foil, and 0.5 mL of distilled water and 4 mL of 3% formic acid were added; the samples were then stirred in a vortex and kept in orbital agitation for 15 min. The absorbance was read at 280 nm in quartz cells after having been centrifuged for 5 min at 6500 rpm and filtered by gravity.

The result was expressed as milligrams of gallic acid equivalent per gram of product (mg GAE/100 g dw) using a calibration line for different quantities of the standard, made from solutions of different concentrations (0–400 µg/mL) of gallic acid with the same treatment as the samples, and left stand in darkness during 15 min at room temperature. Measurement of absorbance at 280 nm was read in a Synergy HTX multi-mode reader spectrum using microplates.

- Q-Hydroxycinnamic acids

The determination of hydroxycinnamic acids was adapted from Bonoli et al., (2004) [17]. Triplicate samples of dry plant material were weighed (1 ± 0.1 mg) in Falcon tubes using a precision balance. The tubes were covered with aluminium foil, and 0.5 mL of distilled water and 4 mL of methanol were added, then stirred in a vortex and kept in orbital agitation for 15 min. The absorbance was read at 320 nm in a Synergy HTX multi-mode reader spectrum using microplates after centrifugation for 5 min at 6500 rpm and filtering by gravity.

The results were expressed as milligrams of ferulic acid equivalent per gram of product (mg FAE/100 g dw) by a calibration line for different quantities of the standard, made from solutions of different concentrations (0–200 µg/mL) of ferulic acid with the same treatment as the samples, and left stand in darkness during 15 min at room temperature. Measurement of absorbance at 320 nm was read in a Synergy HTX multi-mode reader spectrum using microplates.

- Q-Flavonols

The determination of flavonols was adapted from Bonoli et al., (2004) [17]. Triplicate samples of dry plant material were weighed (1 ± 0.1 mg) in Falcon tubes. The tubes were covered with aluminium foil, and then 0.5 mL of distilled water and 4 mL of methanol were added, stirred in a vortex, and kept in orbital agitation for 15 min. The absorbance was read at 370 nm in a Synergy HTX multi-mode reader spectrum using microplates after centrifugation for 5 min at 6500 rpm and filtration by gravity.

The result was expressed as milligrams of quercetin equivalent per gram of product (mg QE/100 g dw) by a calibration line for different quantities of the standard, made from solutions of different concentrations (0–250 µg/mL) of quercetin with the same treatment as the samples, and left stand in darkness during 15 min at room temperature. Measurement of absorbance at 370 nm was read in a Synergy HTX multi-mode reader spectrum using microplates.

- Q-Total monomeric anthocyanins

Total monomeric anthocyanins in dry plant material were determined, according to Giusti and Wrolstad (1996), by the pH difference method [18]. Two buffer solutions were used: 0.025 M KCl at pH 1 and 0.4 M CH_3_CO_2_Na at pH 4.5. An ethanol/water solution (80:20, *v*/*v*) was used as a solvent for both buffers.

After crushing and sifting the sample, approximately 8 mg of freeze-dried fruit epidermis were weighed in Falcon tubes and covered with aluminium foil to avoid light exposure. Subsequently, 10 mL of each buffer were added and kept in circular agitation for 15 min, then centrifuged for 5 min at 7000 rpm, filtered by gravity, and absorbance was measured at 510 nm (λvis-max) and 700 nm (to correct interference), corresponding to the colored oxonium form at pH 1.0 and the colorless hemiketal form at pH 4.5. Results were expressed in mg cyanidin-3-glucoside per 100 g of dry fruit material.

Using the following formulas, the total anthocyanins were calculated:

A = (A _510_ − A _700_) _pH 1.0_ − (A _510_ − A _700_) _pH 4.5_, (1)

Total anthocyanins (mg/L) = (A × MW × DF × 1000)/(ε × 1),(2)

where MW = cyanidin-3-glucoside molecular weight, DF = dilution factor, and ε = molar extinction coefficient.

- In vitro antioxidant capacity

Antioxidant capacities were measured through QUENCHER methodology, using three different assays: Q-DPPH, Q-Folin–Ciocalteu, and Q-FRAP.

The antioxidant capacity was determined by the Q-DPPH method in dry plant material following the methodology proposed by Del Pino-García et al., (2015) [13]. This method is based on the sweeping of Q-DPPH radicals. A solution of 0.1 mM Q-DPPH was mixed with ethanol/water (50:50, *v*/*v*) to obtain an absorbance between 0.75 and 0.80 at 517 nm. Then, each sample of dry plant material was weighed (5 ± 0.1 mg) in triplicate in Falcon tubes, covered with aluminium foil, and 10 mL of the Q-DPPH dilution was added. Afterwards, they were stirred in a vortex and kept under circular agitation for 1 h. After being centrifuged for 5 min at 7000 rpm and filtered, the absorbance at 517 nm was read in a Synergy HTX multi-mode reader spectrum using microplates. Trolox was used as standard to perform a calibration line from different concentrations (0–400 µg/mL) of dissolution of Trolox, mixed with Q-DPPH reagent, and left stand in darkness and room temperature for 1 h; then, absorbance was measured at 517 nm in a Synergy HTX multi-mode reader spectrum using microplates. The results were expressed as milligrams of TE per gram of product (mg TE/100 g dw).

Q-Folin–Ciocalteu was adapted from Slinkard and Singleton (1977) [19]. Each sample of dry plant material was weighed (1 ± 0.1 mg) in triplicate in Falcon tubes, covered with aluminium foil, and 0.8 mL of distilled water and 0.2 mL of Folin–Ciocalteu phenol reagent (Scharlab S.L.) were added and stirred in a vortex. After 5 min of reaction, 4 mL of 0.7 M Na_2_CO_3_ and 5 mL of distilled water were added. After stirring in a vortex and 45 min in orbital agitation, the absorbance was read at 750 nm in a Synergy HTX multi-mode reader spectrum using microplates after centrifugation 10 min at 6500 rpm and filtering. The results were expressed as milligrams of gallic acid equivalent per gram of product (mg GAE/100 g dw) by a calibration line for different quantities of the standard, made from solutions of different concentrations (0–400 µg/mL) of gallic acid with the same treatment as the samples, and left stand in darkness during 1 h at room temperature. Measurement of absorbance at 750 nm was read in a Synergy HTX multi-mode reader spectrum using microplates.

For Q-FRAP analysis, the methodology described by Benzie and Strain (1996) was used for the determination of the Fe (III) reduction activity [20], with modifications from Del Pino-García et al., (2015), at 595 nm [13].

In Falcon tubes covered with aluminium foil, each sample of dry plant material was weighted (1 ± 0.1) in mg using a precision balance (BOECO Germany). Later, 40 mL of FRAP reagent were added and incubated at 37 °C for 30 min with continuous stirring. The absorbance was measured at 593 nm in a Synergy HTX multi-mode reader spectrum using microplates after being centrifuged for 5 min at 7000 rpm and filtered by gravity.

Trolox was used as standard to perform a calibration line from different concentrations (0–250 µg/mL) of Trolox, mixed with FRAP reagent, and left stand at 37 °C for 1 h. Then, absorbance was measured at 595 nm in a Synergy HTX multi-mode reader spectrum using microplates. The results were expressed as milligrams of TE per gram of product (mg TE/100 g dw).

#### 2.2.3. Individual Anthocyanin Profile

- Extraction procedure

The extraction procedure for anthocyanins was adapted from Primo da Silva et al., (2019) [21]. Each sample of dry plant material (1 g) was extracted by a maceration extraction methodology for 1 h, with 30 mL of ethanol/water (80:20, *v*/*v*) acidified with 0.5% HCl. After gravity filtration, the residue was re-extracted with 30 mL of ethanol/water (80:20, *v*/*v*) acidified with 0.5% HCl. To eliminate ethanol, the combined extracts were evaporated using rotavapor at 40 °C and 70 mBar. They were subsequently frozen and lyophilized.

- Identification of anthocyanins in the extracts by HPLC/MS

Anthocyanins were determined in the extracts by HPLC/MS as described by Bastos et al., (2015) [22]. Extraction of 1 g of each sample with ethanol:water (80:20) was made for the identification of the individual anthocyanins profile. The extract obtained was freeze-dried, and 5 mg of the extract were re-dissolved in 1 mL of water and filtered through a 0.22 μm disposable LC filter disc into an amber vial for analysis by HPLC.

The dissolution was injected in HPLC/MS equipment (Dionex Ultimate 3000 UPLC, Thermo Scientific, San Jose, CA, USA) coupled to a diode-array detector (using 520 nm as preferred wavelength) and an electrospray ionization mass spectrometer (Linear Ion Trap LTQ XL, Thermo Scientific) working in positive mode. An AQUA^®^ reverse phase C_18_ column (5 µm, 150 mm × 4.6 mm, Phenomenex) was used at 35 °C for compound separation with gradients previously described [23]. Anthocyanin identification was performed using retention time, UV-VIS, and mass spectra compared to the available standards and literature data; quantification was carried out using a 7-level calibration curve of the standard calibration anthocyanin compound Cyanidin-3-*O*-glucoside (*y* = 103,505*x* + 3 × 10^6^; *r*^2^: 0.9914; LOD: 0.15 g/mL; LOQ: 0.45 g/mL). The quantitative results were expressed in mg/g extract.

#### 2.2.4. Statistical Analysis

All the analyses were carried out in triplicate. Statgraphics Centurion 18.1.16 (64-bit) (Statistical Graphics Corporation, Inc., Rockville, MD, USA) was used for the statistical treatment of the analytical data. The simple ANOVA test and Fisher’s least significant difference (LSD) post hoc test were used to compare pairs of means and determine statistical significance at the *p* < 0.05 level. For anthocyanin phenolic compounds quantification and comparison samples, a Student’s *t*-test was used to determine the significant differences between the two sites, with *p* = 0.05. Additionally, principal component analysis (PCA) was performed among the variables analyzed using the same software.

## 3. Results and Discussion

### 3.1. Characterization of Fresh Fruits

The results of the initial characterization of the whole fruits of *C. monogyna* and *S. aria* in 2021 are provided in Table 2 with total average values, considering the samples by triplicate in both locations (*n* = 12). The natural variability due to geographical and ripening status variations were expected to influence the chemical composition of the fruits because of differences in soil composition and environmental conditions [24].

The average content of water in *C. monogyna* was 74.6% (*w*/*w*), a similar value to those described by Egea et al., (2010) (73.5%) [25]; whereas, in *S. aria*, it was 64.4% (*w*/*w*), higher than the values described by Petkova et al., (2020) (54.5%) [10]. Additionally, there is a significant difference in moisture content between both sites of *S. aria* fruits, possibly due to weather and terrain, which contribute to fruit desiccation.

Regarding °Brix, significant differences were found among the analyzed samples, although fruits of *S. aria* showed, in general, higher values than *C. monogyna*. This parameter is directly related to sugar content and maturity state. Previous studies in *C. monogyna* fruits [8] reported higher °Brix values (19.58) than those found in the present study (0.63), whereas no data were found for *S. aria*.

As shown in Table 2, there were significant differences between the pH and titratable acidity of the fruits among species and sites. The pH increases due to several factors; the main reason is the degradation of organic acids and the accumulation of soluble sugars present in the fruit during the ripening process. pH values were higher for *C. monogyna* fruits compared to Alirezalu et al., (2020) (3.93) [8]; this may be due to genetic differences, environmental conditions, and the ripeness degree of the analyzed fruits. In this sense, it is possible that the samples collected in the present study were more mature than those used by Alirezalu et al., (2020) [8]. The average result for *S. aria* fruits is very similar to those reported by Petkova et al., (2020) (4.22) [10].

### 3.2. Color

The intensity of the pigmentation in fruits depends on several factors, including genetic and environmental factors. The characteristic reddish color of these fruits should be attributed to anthocyanin compounds [25]. Instrumental color measurements of lyophilized and powdered epidermis of the studied fruits through CIELAB color parameters (L*, a*, and b*) were analyzed.

Regarding *C. monogyna* and *S. aria* epidermis color parameters (Table 3), it can be seen that *C. monogyna* always showed higher values of parameter a* (higher intensity of red color) and lower values of b* (lower intensity of yellow–orange color) and L* (brightness). There is a significant difference between the parameters measured in the epidermis from both locations, with the second location yielding more reddish results compared to the first. This could be due to a higher presence of anthocyanins in the fruits of the second location. Additionally, the results differ from those reported by Egea et al., (2010) (L* 34.91, a* 44.12, and b* 19.26) [25] and Alirezalu et al., (2020) (L* 7.37, a* 33.95, and b* 12.55) for the edible parts (pulps and peels without seeds); although, in both cases, the final color is reddish [8]. These differences may be because, in the present study, only the epidermis was analyzed.

Regarding *S. aria* epidermides, they showed lower values of parameter a* (lower intensity of red color) and higher b* (higher intensity of yellow–orange color)) and L* values. There is a significant difference between the parameters depending on the location of the fruits, and no data were found in the literature concerning *S. aria*, although Egea et al., (2010) studied the species *S. domestica*, in which visual color was brown instead of red–orange [25]. Additionally, there is a significant difference between all the parameters from both types of fruits; for that reason, *C. monogyna* has reddish fruits, whereas *S. aria* has red–orange fruits. As mentioned before, this is due to the difference between parameters a* and b*. Regarding the results of *C. monogyna*, having higher values of a* (16.15 and 19.33, respectively) and lower values of b* (20.65 and 15.88, respectively) results in a more reddish color compared to the fruits of *S. aria*, which, on the contrary, have lower values of a* (9.06 and 12.74, respectively) and higher values of b* (29.34 and 29.27, respectively).

### 3.3. Phenolic Compounds Content

The diversity of methodology and standards for quantification used by different authors makes comparison with the results of phenolic compounds from previous studies very difficult. On the one hand, many studies report values of the whole fruits and not the epidermis. On the other hand, the differences in methodology make the comparison of data difficult since the Folin–Ciocalteu method is often used as a measure of TPC; although, nowadays, it is considered more than just a method to measure antioxidant capacity [4].

Several authors determined the TPC by Fast Blue BB in different berries [26,27]. Lester et al., (2012) [27] concluded that the Fast Blue-BB assay provides a higher and more accurate estimate of total phenolics due to its direct reaction with phenolics in strawberry fruits than the current indirect total phenolics Folin–Ciocalteu assay that can be affected by interference from sugars and other compounds [28]. Previous authors performed extractions which could underestimate the content of TPC. while; for the QUENCHER procedure, there are no limitations of solubility, binding to other molecules, etc. In this sense, Palombini et al., (2016) combined Fast Blue BB reagent and the QUENCHER procedure for the first time [16].

The present study used a combination of both methodologies (Fast Blue BB and QUENCHER), which has not been applied to this type of sample before and allows improved quantification of TPC. As shown in Table 4, the *C. monogyna* epidermis was a richer source of phenolic compounds than *S. aria*, providing more than double these compounds. Hawthorn presented an average value of total phenolic compounds (TPC) of 6178 mg gallic acid equivalent (GAE) per 100 g dw (dry weight), as measured by Q-Fast Blue BB. In the case of *C. monogyna*, hydroxybenzoic acids were predominant (2871 mg GAE/100 g dw), followed by flavonols (771 mg QE/100 g dw) and hydroxycinnamic acids (610 mg FAE/100 g dw). Total monomeric anthocyanins were the minor family, with an average value of 252 mg cyanindin-3-glucoside/100 g dw.

Phenolic compound families showed a different profile in *S. aria*, where hydroxybenzoic acids presented the highest content (942 mg GAE/g dw), followed by hydroxycinnamic acids (558 mg FAE/g dw) and flavonols (317 mg QE/g dw). Additionally, total monomeric anthocyanins were the minor phenolic compound (34 mg cyanindin-3-glucoside/100 g dw) found in this fruit peel. There were statistically significant (*p* < 0.05) differences between fruits collected in different sites regarding all the analyzed parameters. Fruits from site 1 presented higher content of total phenolic compounds (4057 vs. 1831 mg GAE/g dw) than fruits from site 2, with a similar result for hydroxybenzoic acids (894 vs. 991 mg GAE/100 g dw), hydroxycinnamic acids (615 vs. 501 mg FAE/100 g dw), and flavonols (242 vs. 393 mg QE/100 g dw), and lower content of total monomeric anthocyanidins (16 vs. 52 mg cyanindin-3-glucoside/100 g dw).

The differences found within species may be due to natural variability, which is often described in plant samples [29]. Despite the significant difference between sites, *C. monogyna* fruit peels present higher values of TPC and all the phenolic families studied compared to *S. aria*, including monomeric anthocyanins. Although monomeric anthocyanins may be minor compared to other phenolic compounds, they are sufficient to provide color. Comparing fruits of both species and despite variations among sites, *C. monogyna* presented the highest levels of monomeric anthocyanins, which agrees with both visual color and higher values of CIELAB a* parameter, reflecting the higher intensity of red color in hawthorn fruits. It is important to note that natural variability is often described in plant samples. In addition, other studies have shown that environmental factors can influence the phenolic composition and antioxidant capacity of *C. monogyna* and *S. aria* [29].

### 3.4. In Vitro Antioxidant Capacity

When applying QUENCHER methodology to the analysis of the epidermis of the fruits analyzed, it was obtained that *C. monogyna fruits*, which showed the highest phenolic compound content, were also found to have the highest antioxidant capacity measured by the Q-DPPH, Q-Folin–Ciocalteu, and Q-FRAP methods (Table 5). In our study, three methods, based on two different mechanisms, SET (single electron transfer) and HAT (hydrogen atom transfer), were used to evaluate the antioxidant activity of *C. monogyna* and *S. aria* fruits collected from the Iberian Peninsula [10]. In this case, Q-DPPH was used to evaluate the SET mechanism, whereas Q-Folin–Ciocalteu and Q-FRAP were used to evaluate the HAT mechanism.

In this study, *C. monogyna* fruits were analyzed by the Q-Folin–Ciocalteu method, and an average result of 3740 mg GAE per 100 g of dry weight was obtained. These values were higher than those reported by Ruiz-Rodríguez et al., (2014) (2721 mg GAE/100 g dw) for freeze-dried seedless fruits [4]. In addition, Egea et al., (2010) (217 mg GAE/100 g dw) and Alirezalu et al., (2020) (3585 mg GAE/100 g dw) reported lower values for fruit extracts [8,24]. This assay is based on the principle of oxidation of phenolic compounds by the Folin–Ciocalteu reagent, which is a mixture of phosphotungstic and molybdic acids. It is important to mention that it may interact with other reducing compounds such as sugars, ascorbic acid, etc. The Q-DPPH method showed 874 mg Trolox equivalent (TE) per 100 g (dw). This value is lower than Moldovan et al., (2021) reported (3434 mg TE/100 g of dw) because, in their study, the antioxidant activity was determined in the extract of the whole fruit [30]. Respecting the Q-FRAP method, an average result of 13.781 mg Trolox equivalent per 100 g of dry weight was obtained; this value was higher than Moldovan et al., (2021) reported (7434 mg TE/100 g dw), despite using fruit extracts [30]. In the present study, the antioxidant compounds present in the epidermis of *C. monogyna* may have a greater capacity to reduce the ferric cation (Fe^3+^) in the FRAP assay than to neutralize the DPPH radical in the DPPH assay. This could be due to the predominant presence of strong reducing compounds in the epidermis, such as ascorbic acid or tannins, which exhibit high activity in the FRAP assay. Furthermore, the FRAP assay can be more sensitive and accurately detect changes in the antioxidant capacity of the lyophilized fruit’s epidermis samples. This could result in a higher measurement in the FRAP assay, even if the antioxidant activity measured by the DPPH assay is lower. On the other hand, the reaction mechanism between the antioxidant and DPPH depends on the conformational structure of the antioxidant. The steric accessibility of the DPPH radical is an important determining factor in the reaction, as small molecules that have better access to the radical site have a relatively higher antioxidant capacity [31]. In this regard, it is important to note that anthocyanins are anthocyanidins bound to one or more sugar residues. The most common ones are glucose, galactose, xylose, rhamnose, and arabinose; disaccharides such as rutinose, sophorose, and sambubiose, or trisaccharides such as 2-xylosylrutinose and glucurosylrutinose, are also frequent [31]. This glycosylation confers greater stability to them and increases their solubility in water compared to anthocyanidins; however, it could create steric hindrance that justifies a lower interaction with DPPH and, therefore, a lower result using the DPPH method [31].

The epidermis of the fruits of *S. aria* analyzed in this study showed a value of 860 mg gallic acid equivalent per 100 g of dry weight for Q-Folin–Ciocalteu determination (Table 5), similar values to those reported by Tahirovic et al., (2019) (702 mg GAE/100 g dw) for whole fruits [32], and lower values than those reported by Petkova et al., (2020) (7125 mg GAE/100 g dw) for seedless fruits [10]. Applying the Q-DPPH method and the Q-FRAP method, the values for peel fruits of *S. aria* were 873 mg Trolox equivalent per 100 g of dry weight and 6086 mg Trolox equivalent per 100 g of dry weight, respectively. These are the first results on the antioxidant capacity in *S. aria* epidermides, which have not been reported before.

As can be observed, *C. monogyna* presents higher antioxidant capacity measured by Q-Folin–Ciocalteu and Q-FRAP than *S. aria*, while for Q-DPPH, the results are similar in both fruits. This can be related to the different mechanisms of antioxidant action measured by different methods. On the one hand, Q-DPPH is an electronic transfer method based on the neutralization of the artificial radical DPPH. Steric accessibility to this radical is an important determinant of the reaction since small molecules that have better access to the radical site have a relatively higher antioxidant capacity [33]. On the other hand, both Q-Folin–Ciocalteu and Q-FRAP evaluate antioxidant capacity through an electronic transfer mechanism based on the reducing capacity of metal ions and in the case of Q-FRAP, ferric ions. However, the reagent Folin–Ciocalteu is not specific to polyphenols [31]. It can also be reduced with other substances such as ascorbic acid, sugars, or copper ions; in fact, phenols react only in basic medium [34]. Regarding Q-FRAP, it is carried out in an acidic medium at pH 3.6, which is too low compared to the physiological one [33], and this implies that many non-antioxidant compounds could reduce it [23]. These factors make the evaluation of antioxidant capacity through different types of assays necessary.

To conclude, there are numerous studies that link the content of phenolic compounds with antioxidant activity, which can explain the different results regarding the content of different phenolic compounds [35,36,37,38].

### 3.5. Characterization of Anthocyanins in Extracts Obtained from the Epidermis of the Fruits

**Figure 2:**
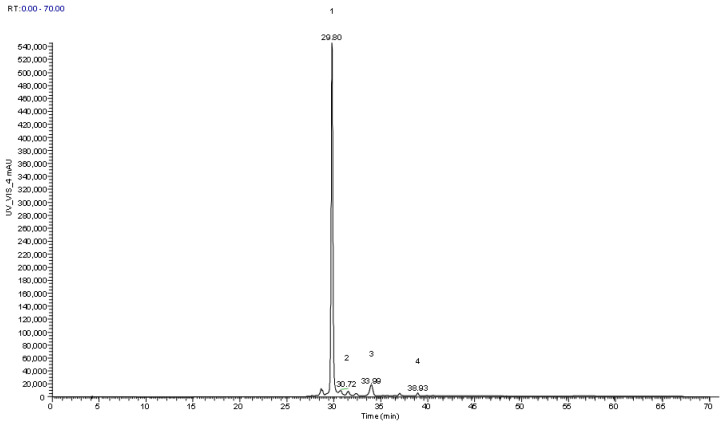
Chromatographic profile of the anthocyanin compounds found in *Crataegus monogyna* extracts, recorded at 520 nm (1: cyanidin-*O*-hexoxide; 2: cyanidin-3-*O*-glucoside; 3: peonidin-*O*-hexoxide; 4: cyanidin derivative).

**Figure 3:**
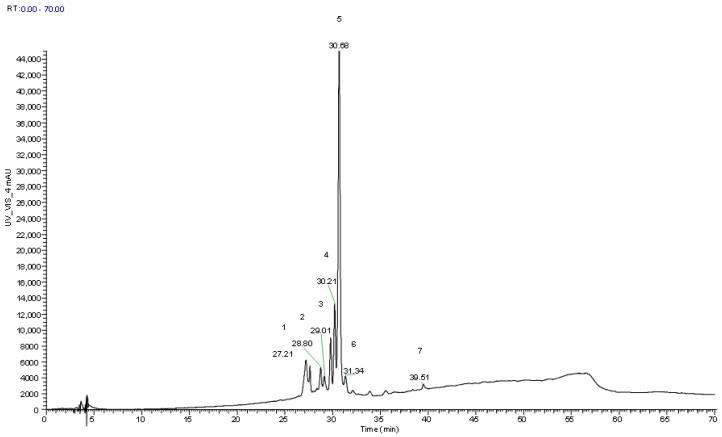
Chromatographic profile of the anthocyanin compounds found in *Sorbus aria* extracts, recorded al 520 nm (1, 2, and 3: cyanidin-*O*-hexosyl-*O*-hexoxide; 4: cyanidin-*O*-hexosyl-*O*-pentoxide; 5: cyanidin-3-*O*-glucoside; 6: cyanidin-*O*-deoxyhexosyl-pentoxide; 7: cyanidin derivate).

As explained before, hydroalcoholic extraction of the epidermis of the fruits of *C. monogyna* and *S.aria* was made, followed by clean-up and HPLC/MS analysis, with the purpose of identifying anthocyanin phenolic compounds at 520 nm (Table 6 and Table 7, respectively) by comparing the retention times, maximum absorption in the UV visible region, and spectral data with authentic standards and scientific published data in similar fruits (Figure 2 and Figure 3). Peaks 2 ^cm^/5^sa^ ([H]^+^ at *m*/*z* 449) were identified by comparing their retention time and UV spectra with the authentic standard compound (cyanidin-3-*O*-glucoside). Peak 1cm presented the same spectral behavior regarding UV absorption and mass, being tentatively identified as cyanidin-*O*-hexoxide. Three similar cyanidin O-glycosides were also found, peaks 1 ^sa^, 2 ^sa^, and 3^sa^, that presented a protonated ion [H]^+^ at *m*/*z* 611, and two main MS^2^ fragments at *m*/*z* 449 (162 u) and 287 (162 u), that corresponded to the loss of two consecutive hexosyl units, being tentatively identified as cyanidin-*O*-hexosyl-*O*-hexoxide. Peaks 4 ^sa^ ([H]^+^ at *m*/*z* 581) and 6 ^sa^ ([H]^+^ at *m*/*z* 565), both presented one unique MS^2^ fragment of *m*/*z* 287, cyanidin aglycone, that indicated the joint loss of hexosyl-pentosyl ([H-162-132]^+^) units and deoxyhexosyl-pentosyl [H-146-132]^+^ units, respectively, being tentatively identified as cyanidin-*O*-hexosyl-*O*-pentoxide and cyanidin-*O*-deoxyhexosyl-pentoxide, respectively. One last cyanidin derivative was also found in both samples, peaks 4^cm^ and 7^sa^, that presented a protonated ion [H]^+^ at *m*/*z* 557 and two main MS^2^ fragments at *m*/*z* 395 and 287, but at that moment, it was not possible to identify the moiety linked to the aglycone of cyanidin.

Finally, one O-glycosylated peonidin derivative found in *C. monogyna* samples, peak 3^cm^ ([H]^+^ at *m*/*z* 463), was tentatively identified as peonidin-*O*-hexoxide.

In the analyzed samples, major compounds were identified and quantified according to their retention times and absorption maxima in the UV-Vis spectra. As shown in Table 6 and Table 7, cyanidin-*O*-hexoxide is the predominant anthocyanin in *C. monogyna* epidermis extracts (6.62–9.88 mg/g extract), and peonidin-*O*-hexoxide and cyanidin derivatives were also identified (Table 6). This result is higher than the results reported by Mraihi et al., (2015) for cyanidin-*O*-hexoxide (0.49 mg/g extract) in *C. monogyna* fruits [39].

In the case of *S. aria* epidermis extract, cyanidin-*O*-hexosyl-*O*-hexoxide, cyanidin-*O*-hexosyl-pentoxide, cyanidin-*O*-hexoxide, cyanidin-*O*-deoxyhexosyl-pentoxide, and cyanidin derivate were identified (Table 7). As in hawthorn fruits, the major anthocyanin was cyanidin-*O*-hexoxide (0.65–5.15 mg/g extract).

Isomers of the same compounds with different retention times were identified in both species. Two cyanidin-*O*-hexoxide isomers were identified in the case of *C. monogyna*, and in the case of *S. aria*, different isomers of cyanidin-*O*-hexosyl-*O*-hexoxide and cyanidin-*O*-hexosyl-pentoxide were identified.

### 3.6. Principal Component Analysis (PCA)

**Figure 4:**
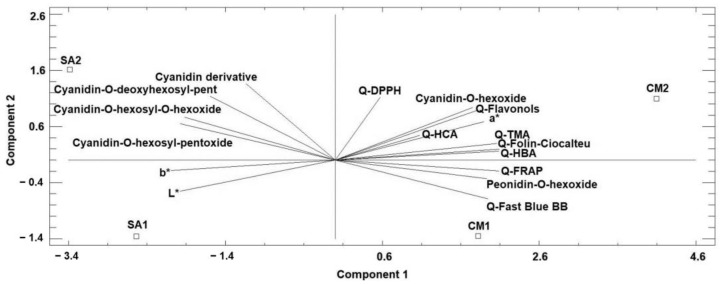
Principal component analysis (PCA) projection of two principal components on CIELAB parameters, total phenolic compounds, antioxidant activity, and anthocyanins profile. Samples letters: CM1: *Crataegus monogyna* site 1; CM2: *Crataegus monogyna* site 2; SA1: *Sorbus aria* site 1; SA2: *Sorbus aria* site 2.

To globally interpret all the analytical parameters obtained: color, TPC, in vitro antioxidant capacity, and anthocyanins characterization, a study of principal component analysis was performed, as shown in Figure 4, to show the difference between the analyzed species.

The PCA analysis explained 89.05% of the total variance. The PCA biplot clearly differentiates *C. monogyna* from *S. aria* fruits.

The first principal component (74.38% of the total variance) was positively correlated with CIELAB a* parameter (0.25), Q-Fast Blue BB (0.26), Q-Hydroxybenzoic acids (0.28), Q-Flavonols (0.24), Q-Total monomeric anthocyanins (0.27), Q-Folin–Ciocalteu (0.28), Q-FRAP (0.28), cyanidin-*O*-hexoxide (0.23), and peonidin-*O*-hexoxide (0.26), while it correlated with Q-Hydroxycinnamic acids (0.14) and Q-DPPH (0.08) to a minor degree; it was negatively correlated with CIELAB b* and L* parameters (−0.28 and −0.26, respectively), and the individual anthocyanins identified were cyanidin derivative (−0.15), cyanidin-*O*-hexosyl-*O*-hexoxide (−0.26), cyanidin-*O*-hexosyl-pentoxide (−0.26), and cyanidin-*O*-deoxyhexosyl-pentoxide (−0.21).

The second principal component (14.67% of the total variance) was strongly correlated with CIELAB a* parameter (0.23), Q-Flavonols (0.3), Q-DPPH (0.38), cyanidin-*O*-hexoxide (0.31), cyanidin derivative (0.46), cyanidin-*O*-hexosyl-*O*-hexoxide (0.26), cyanidin-*O*-hexosyl-pentoxide (0.22), and cyanidin-*O*-deoxyhexosyl-pentoxide (0.38), while it was positively correlated with Q-Hydroxybenzoic acids (0.05), Q-Hydroxycinnamic acids (0.15), Q-Total monomeric anthocyanins (0.10), and Q-Folin–Ciocalteu (0.07) to a minor degree; it was negatively correlated with Q-Fast Blue BB (−0.23), Q-FRAP (−0.07), CIELAB b* and L* parameters (−0.06 and −0.19, respectively), and peonidin-*O*-hexoxide (−0.11).

The CM2 sample was most positively characterized by the first component (4.09) and less by the second component (1.09), while CM1 was positively characterized by the first component (1.82) and negatively characterized by the second component (−1.35). The SA1 sample was negatively characterized by the first and second components (−2.53 and −1.36, respectively), while the SA2 sample was positively characterized by the second component (1.61) and negatively characterized by the first component (−3.38).

Despite the natural variability found among fruits collected in different sites, principal component 1 clearly differentiated both species.

Hawthorn fruits were differentiated by their higher levels of TPC (Q-Fast Blue BB), the families of phenolics analyzed (hydroxybenzoic acids, hydroxycinnamic acids, flavonols, and total monomeric anthocyanins), and their in vitro antioxidant capacity measured by all the methods applied; CIELAB a* parameter and individual compounds cyanidin-*O*-hexoxide and peonidin-*O*-hexoxide were not found in *S. aria* and are, therefore, characteristic of *C. monogyna* peel fruit.

On the other hand, whitebeam fruit peels were mostly characterized by CIELAB b* and L* parameters, in agreement with the higher intensity of red–orange color, and SA2 by the content of cyanidin-*O*-hexosyl-*O*-hexoxide, cyanidin-*O*-hexosyl-pentoxide, cyanidin-*O*-deoxyhexosyl-pentoxide, and cyanidin derivate.

## 4. Conclusions

In the current study, the fruit epidermides of hawthorn (*C. monogyna*) and whitebeam (*S. aria*) were characterized as a natural source of phytochemicals such as anthocyanins, phenolic acids, and flavonoids. In the case of *S. aria*, the data provided are the first report of fruits from the Iberian Peninsula. The antioxidant properties of the fruits demonstrated their potential for food preparation with potential beneficial effects, and the anthocyanin content could be used as a natural dye in the food industry. *C. monogyna* has a higher content of all phenolic compounds, being the majority of hydroxybenzoic acids, followed by flavonols and hydroxycinnamic acids and anthocyanins. Cyanidin-*O*-hexoxide was the major one, correlating with higher values of a* parameter (related to the higher intensity of the reddish color of the epidermis of these fruits). On the other hand, *S. aria* epidermides were characterized for the first time, showing a higher content of hydroxybenzoic acids, followed by hydroxycinnamic acids and flavonols, and anthocyanins, which are in very little quantity in the epidermides of these fruits; different cyanidin derivatives are found in these fruits, providing a yellow–orange color, related to b* parameter. From these results, the fruits investigated, particularly the *C. monogyna* epidermis, could be promising ingredients for the food industry for either their coloring or antioxidant potential.

## Figures and Tables

**Table 1:** Sites where the studied fruits were collected.

	Site 1	Site 2
*Crataegus monogyna* Jacq.		
Municipality	“Finca El Encín”,	Monte de Valdelatas
	Sotos del Henares	
Province	Madrid (Spain)	Madrid (Spain)
Latitude	40.517947	40.541579
Longitude	−3.297917	−3.683101
*Sorbus aria* (L.) Crantz		
Municipality	Zarzuela de Galve,	Puerto de la Quesera,
	Valverde de los Arroyos	Riofrío de Riaza
Province	Guadalajara (Spain)	Segovia (Spain)
Latitude	41.155864	41.219553
Longitude	−3.248964	−3.418064

**Table 2:** Characterization of the whole fruits of *Crataegus monogyna* Jacq. and *Sorbus aria* (L.) Crantz.

	Moisture (g/100 g fw)	°Brix	pH	Titratable Acidity (meq of NaOH/100 g fw)	Ripening Index
*Crataegus monogyna* Jacq. (Hawthom)					
Site 1 Site 2	75.1 ± 0.3 ^c^ 74.2 ± 0.1 ^c^	5.33 ± 0.06 ^a^ 7.33 ± 0.06 ^b^	4.55 ± 0.08 ^c^ 4.28 ± 0.02 ^b^	13.50 ± 0.25 ^c^ 8.68 ± 0.42 ^a^	0.004 ^a^ 0.008 ^b^
Average	74.6	6.33	4.42	11.09	0.006
Range	74.1–75.4	5.0–8.0	4.26–4.61	8.26–13.76	0.004–0.009
*Sorbus aria* (L.) Crantz (Whitebeam)					
Site 1 Site 2	63.2 ± 0.34 ^a^ 65.6 ± 1.48 ^b^	14.33 ± 0.06 ^d^ 9.33 ± 0.06 ^c^	4.26 ± 0.01 ^b^ 4.07 ± 0.01 ^a^	10.25 ± 0.3 ^b^ 8.69 ± 1.13 ^a^	0.014 ^d^ 0.011 ^c^
Average	64.4	11.83	4.17	9.47	0.013
Range	62.9–67.2	9.0–15.0	4.06–4.26	7.71–10.58	0.009–0.014

Values expressed as mean ± standard deviation (SD), *n* = 3; fw (fresh weight). Different letters in the same column mean statistically significant differences (*p* < 0.05).

**Table 3:** Color of *Crataegus monogyna* Jacq. and *Sorbus aria* (L.) Crantz epidermis (average parameters of dry plant material).

	L*	a*	b*	RGB
*Crataegus monogyna* Jacq. (Hawthom)				
Site 1	57.70 ± 0.05 ^b^	16.15 ± 0.02 ^c^	20.65 ± 0.02 ^b^	RGB (177, 127, 103)
Site 2	51.37 ± 0.02 ^a^	19.33 ± 0.05 ^d^	15.88 ± 0.05 ^a^	RGB (162, 110, 96)
*Sorbus aria* (L.) Crantz (Whitebeam)				
Site 1	71.02 ± 0.22 ^d^	9.06 ± 0.14 ^a^	29.34 ± 0.08 ^c^	RGB (207, 167, 121)
Site 2	65.72 ± 0.07 ^c^	12.74 ± 0.04 ^b^	29.27 ± 0.04 ^c^	RGB (198, 150, 108)

L*: luminosity, a*: red–green; b*: yellow–blue. Values expressed as mean ± standard deviation (SD). Different letters in the same column mean statistically significant differences (*p* < 0.05).

**Table 4:** Total phenolic compounds and phenolic families by QUENCHER methodology (Q-Fast Blue BB, Q-Hydroxybenzoic acids, Q-Hydroxycinnamic acid, and Q-Flavonols) of *Crataegus monogyna* Jacq. and *Sorbus aria* (L.) Crantz epidermis.

	Q-Fast Blue BB (mg GAE/100 g dw)	Q-HBA (mg GAE/100 g dw)	Q-HCA (mg FAE/100 g dw)	Q-Flavonols (mg QE/100 g dw)	Q-TMA (mg cya-3-glu/100 g dw)
*Crataegus monogyna* Jacq. (Hawthom)					
Site 1 Site 2	5965.2 ± 397.1 ^c^ 6391.5 ± 228.3 ^d^	2538.7 ± 217.3 ^b^ 3202.6 ± 87.5 ^c^	481.3 ± 40.2 ^a^ 739.3 ± 66.6 ^c^	491.9 ± 30.9 ^c^ 1050.9 ± 102.0 ^d^	211.6 ± 6.7 ^c^ 291.8 ± 13.2 ^d^
Average	6178.4	2870.6	610.3	771.4	251.7
Range	6636.8–5551.9	3298.8–2312.5	841.0–445.1	1177.6–449.6	304.9–204.4
*Sorbus aria* (L.) Crantz (Whitebeam)					
Site 1 Site 2	4056.8 ± 160.3 ^b^ 1831.1 ± 115.2 ^a^	894.0 ± 61.3 ^a^ 990.7 ± 36.1 ^a^	615.0 ± 42.4 ^b^ 501.1 ± 43.8 ^a^	242.1 ± 17.9 ^a^ 392.5 ± 34.9 ^b^	15.5 ± 1.1 ^a^ 51.9 ± 2.2 ^b^
Average	2943.9	942.3	558.1	317.3	33.7
Range	4234.0–1701.7	1022.9–839.3	653.1–432.1	440.8–205.2	53.9–14.6

Values expressed as mean ± standard deviation (SD), *n* = 3; dw (dry weight), GAE (gallic acid equivalent), FAE (ferulic acid equivalent), QE (quercetin equivalent), Q-HBA (Q-Hydroxybenzoic acids), Q-HCA (Q-Hydroxycinnamic acids), and Q-TMA (Q-Total Monomeric Anthocyanins). Different letters in the same column mean statistically significant differences (*p* < 0.05).

**Table 5:** In vitro antioxidant capacity by QUENCHER methodology of *Crataegus monogyna* Jacq. and *Sorbus aria* (L.) Crantz epidermis fruit.

	Q-DPPH (mg TE/100 g dw)	Q-Folin–Ciocalteu (mg GAE/100 g dw)	Q-FRAP (mg TE/100 g dw)
*Crataegus monogyna* Jacq. (Hawthom)			
Site 1 Site 2	801.1 ± 30.3 ^a^ 947.2 ± 42.6 ^c^	3036.6 ± 112.1 ^b^ 4443.2 ± 157.7 ^c^	13781.1 ± 639.7 ^c^ 15,350.7 ± 825.8 ^d^
Average	874.2	3739.9	13781.1
Range	730.1–1008.3	2863.0–4648.7	12,800.6–16,538.6
*Sorbus aria* (L.) Crantz (Whitebeam)			
Site 1 Site 2	876.9 ± 37.9 ^b^ 869.8 ± 36.6 ^b^	897.5 ± 27.9 ^a^ 822.5 ± 22.0 ^a^	6823.2 ± 120.9 ^b^ 5348.2 ± 335.6 ^a^
Average	873.4	860.0	6085.7
Range	812.8–921.8	779.9–944.6	5016–7022.1

Values expressed as mean ± standard deviation (SD), dw (dry weight), TE (Trolox equivalent), and GAE (gallic acid equivalent). Different letters in the same column mean statistically significant differences (*p* < 0.05).

**Table 6:** Retention time (Rt), wavelengths of maximum absorption in the visible region (λmax), mass spectral data, identification, and quantification of anthocyanins compounds in *Crataegus monogyna* Jacq. Extracts (mg/g dw).

Peak	Rt (min)	λ_máx_ (nm)	[M]^+^ (*m*/*z*)	MS^2^(*m*/*z*)	Tentative Identification	Quantification (mg/g dw)		*p*-Value
						Site 1	Site 2	
1^cm^	29.8	516	449	287 (100)	Cyanidin-*O*-hexoxide	6.63 ± 0.01	9.876 ± 0.005	<0.001
2^cm^	30.8	517	449	287 (100)	Cyanidin-3-*O*-glucoside	0.20 ± 0.01	0.393 ± 0.005	<0.001
3^cm^	34.0	516	463	301 (100)	Peonidin-*O*-hexoxide	0.355 ± 0.005	0.294 ± 0.002	<0.001
4^cm^	39.0	522	557	395 (100), 287 (10)	Cyanidin derivative	0.087 ± 0.001	0.088 ± 0.001	0.001
					Total anthocyanins	7.27 ± 0.02	10.652 ± 0.002	<0.001

**Table 7:** Retention time (Rt), wavelengths of maximum absorption in the visible region (λmax), mass spectral data, identification, and quantification of anthocyanins compounds in *Sorbus aria* (L.) Crantz extracts (mg/g dw).

Peak	Rt (min)	λ_máx_ (nm)	[M]^+^ (*m*/*z*)	MS^2^(*m*/*z*)	Tentative Identification	Quantification (mg/g dw)		*p*-Value
						Site 1	Site 2	
1^sa^	27.2	513	611	449 (98), 287 (100)	Cyanidin-*O*-hexoyl-*O*-hexoxide	0.15 ± 0.00	Not detected	-
2^sa^	28.8	519	611	449 (12), 287 (100)	Cyanidin-*O*-hexosyl-*O*-hexoxide	0.08 ± 0.00	0.32 ± 0.00	<0.001
3^sa^	29.1	515	611	449 (11), 287 (100)	Cyanidin-*O*-hexosyl-*O*-hexox ide	0.06 ± 0.00	0.25 ± 0.01	<0.001
4^sa^	30.2	515	581	287 (100)	Cyanidin-*O*-hexosyl-*O*-pentoxide	0.15 ± 0.00	Not detected	-
5^sa^	30.7	515	449	287 (100)	Cyanidin-3-*O*-glucoside	0.65 ± 0.00	5.13 ± 0.02	<0.001
6^sa^	31.3	515	565	287 (100)	Cyanidin-*O*-deoxyhexosyl-pentoxide	0.08 ± 0.00	0.43 ± 0.00	<0.001
7^sa^	39.5	523	557	395 (100), 287 (5)	Cyanidin derivate	0.06 ± 0.00	0.31 ± 0.00	<0.001
					Total anthocyanins	1.24 ± 0.01	6.43 ± 0.01	<0.001

## Data Availability

The data used to support the findings of this study can be made available by the corresponding author upon request.
